# An integer-order SIS epidemic model having variable population and fear effect: comparing the stability with fractional order

**DOI:** 10.1186/s42787-022-00153-y

**Published:** 2022-09-30

**Authors:** Manisha Mukherjee, Biswajit Mondal

**Affiliations:** Department of Mathematics, Raja N.L.Khan Women’s College, Midnapore, 721102 India

**Keywords:** SIS model, Fear effect, Stability, Bifurcation, Fractional-order system

## Abstract

This paper investigates the dynamics of an integer-order and fractional-order SIS epidemic model with birth in both susceptible and infected populations, constant recruitment, and the effect of fear levels due to infectious diseases. The existence, uniqueness, non-negativity, and boundedness of the solutions for both proposed models have been discussed. We have established the existence of various equilibrium points and derived sufficient conditions that ensure the local stability under two cases in both integer- and fractional-order models. Global stability has been vindicated using Dulac–Bendixson criterion in the integer-order model. The forward transcritical bifurcation near the disease-free equilibrium has been investigated. The effect of fear level on infected density has also been observed. We have done numerical simulation by MATLAB to verify the theoretical results, found the impact of fear level on the dynamic behaviour of the infected population, and obtained a bifurcation diagram concerning the constant recruitment and fear level. Finally, we have compared the stability of the population in integer and fractional-order systems.

## Introduction

In epidemiology, the study of the infectious disease transmission mechanism into communities, its impact, and the forecast of the future direction of an outbreak, along with the formulation of different optimal policies to control an epidemic, are presented through mathematical models. Hamer formulated a discrete-time model to analyse the recurrence of the measles epidemic [1] in 1906. In 1911, the first deterministic epidemic model to predict the transmission of malaria had been developed by Ross [2]. In 1927, Kermack and Mckendrick [3] formulated an SIR epidemic model to study the spread of infectious disease and also suggested a threshold theory to determine either epidemic or not. An epidemiological model is called an SIS model if the susceptible class remains susceptible even after its recovery from infection. Models of SIS type are appropriate for gonorrhoea and some other bacterial agent diseases in humans [4]. Models of SIS type have also been used for Chagas disease [5], Section 3.5–3.6] and Rocky Mountain spotted fever [4], Section 2.13]. Zhang and Sun [6] discussed the stability analysis of the SIS model with feedback mechanism on the network. El-Saka [7] has developed and analysed an SIS model with bilinear incidence rate and varying population dynamics. He has formulated the epidemic model using differential equations based on the Caputo derivative. Zhang et al. [8] have discussed a stochastic SIS with double epidemic diseases driven by Levy jumps. Liu et al. [9], Wang et al. [10], and Huo and Zhao [11] have formulated SIS and SIR models on complex networks with degree co-relation and linear treatment.

Most epidemiological models have been developed with the disease that spreads quickly (i.e. in less than one year) through the population. The total population size remains constant over the infection period. But when modelling is being developed, with a disease over more than one year, natural births, deaths, and immigration are to be incorporated into the dynamics. Under such consideration, the population may or may not change due to natural births and immigration, which are approximately balanced by disease-related death or not. There are many diseases in which the disease-related death rate radically decreases the population [12]. So the population must be assumed to be variable and a function of time. Two types of disease transmissions occur in epidemiology, horizontal and vertical, where a new infant is not infected or infected by the disease of the mother populations, respectively. Many SIS models have been investigated with variable population considering constant recruitment or natural births of susceptible or susceptible and infected or both recruitment and birth. ZHOU [13] has developed an SIS epidemic model assuming that the constant recruitment, births, and natural deaths co-occur in the host population. Ma [14] has studied an SIS epidemic model with varying population sizes and vaccination. El-Saka [6] has developed and analysed an SIS model with bilinear incidence rate and varying population dynamics.

The changes in human behaviour during the epidemic brought about by infectious diseases have been identified as a dominating factor in epidemics and discussed in the literature [15–18]. The previous epidemic outbreak, such as severe acute respiratory syndrome (SARS) [15, 16] in 2003 and the Middle East respiratory syndrome coronavirus (MERS-CoV) in 2012. Such reactions have been noticed due to the pneumonic plague, severe acute respiratory syndrome (SARS-2, COVID-19), etc., in 2019. Johnston and Warkentin [19] have identified that fear, a common emotional expression of individuals during disease outbreaks, can initiate human protection motivation. In 1965, Geer [20] first proposed the concept and measure of the fear factor, which is the result of external stimuli. Due to such fear of infectious disease, susceptible population has an anti-infectious behaviour. Such behaviour may affect the rate of the infected population and decrease the infected population. This phenomenon can be incorporated by multiplying the total infected population at any instant by the factor [21, 22].

$f(k, I) = \frac{1}{(1+ kI)}$ , where *k* is the level of fear. The function *f*(*k*, *I*) has the properties that

(i) $$f(0, I) = 1$$ and $$f(k, 0) = 1$$
(ii) $$\lim _{k\rightarrow \infty }f(k,I)=0$$ and $$\lim _{I\rightarrow \infty }f(k,I)=0$$
(iii) $$\frac{\partial f}{\partial k}<0$$ and $$\frac{\partial f}{\partial I}<0$$

Taking into account anti-infectious behaviour because of fear, the total infected population $\frac{\beta S I}{(1+kI)}$ is similar to the nonlinear incidence rate in the existing epidemic model. So now we can identify the parameter *k* in nonlinear or saturated incidence rate as the fear factor parameter. Due to incorporating the infectious disease-related fear factor or inhibition effect for the behavioural change of susceptible population in the model, we have considered the saturated or nonlinear incidence rate of the form $\frac{\beta S I}{(1+kI)}$. This rate tends to a saturation level when it gets large, where $\beta I$ represents the infection force, when the disease is entering a fully susceptible population, and $\frac{1}{(1+kI)}$ measures the inhibition effect due to the increase in infected population or the crowding effect of the infective individuals[23]. Cappasso and Serio [24] introduced saturated incidence rate $\frac{\beta SI}{1+\beta \delta I}$ ($\delta >0$) for the cholera epidemic model where the infection force for a large number of infective populations may decrease as the number of infective individuals increases.

The fractional-order differential equation describes a system that depends on the present and past state of the system, and it contains a memory kernel or memory function. But integer-order differential equation describes a system with the present-state only. The fear effect resulting from individual experience (i.e. past state) about the harmful side of the phenomenon is incorporated. Using the fractional-order differential instead of the integer-order differential is reasonable. The fractional-order SIS has been formulated with constant population size and vaccination [25] and variable population size [7]. In both the papers, the existence and the stability of different equilibrium points were studied in 2014. In 2016, a fractional-order SIR model with constant recruitment [26] by discrete model and an SIS model with a constant rate of recruitment and variable population [27] have been developed and analysed by Caputo-type fractional-order derivative. A fractional-order SIS epidemic model has been formulated and discussed by variational iteration and the Euler method.

In this paper, we have investigated the dynamics of an integer-order and fractional-order SIS epidemic model with birth in susceptible and infected populations, along with constant recruitment and fear effect due to infectious disease. According to the best of our knowledge, for the first time, we have studied the fear effect on infected population density under different conditions in our developed model. In this endeavour, we have discussed two different cases (i)$r<\mu$, i.e. the birth rate is less than the natural death rate, and (ii) $r>\mu$, i.e. the birth rate is greater than the natural death rate. Considering the case $r<\mu$, dynamics of a stochastic SIS epidemic model with saturated incidence rate and the variable population is developed by C.Chen and Y.Kang [28], but none has discussed the case when $r>\mu$. We have established the existence of various equilibrium points and derived sufficient conditions that ensure the local and global stability that has been vindicated by using the Dulac–Bendixson criterion in the integer-order model. The forward transcritical bifurcation near the disease-free equilibrium point and the condition under which this occurs have been investigated. The dynamical behaviour of the infected density due to the effect of fear level has been studied. We have done numerical simulation by MATLAB software to verify the theoretical results and find the effect of fear level parameters on the dynamic behaviour of the infected population and obtain bifurcation diagram concerning recruitment and fear level. Finally, we have compared the stability of the model in integer- and fractional-order systems.

The rest part of the paper has been organized as follows: In “Preliminaries” section, the preliminary concept of fractional calculus is explained. In “Model Formulation” section, we give model formulation. “Existence and uniqueness of solutions for integer and fractional order system” and “Nonnegativity and boundedness of solutions for integer and fractional order system” sections describe the uniqueness and boundedness of the solutions of both systems. We have derived the existence of the equilibrium point and reproduction number in “Existence of equilibrium points for the integer-order model” and “Existence of the equilibrium points for fractional-order model” section. In “Local stability analysis at different equilibrium points of integer-order system model” and “Local stability analysis at different equilibrium points for fractional order model” section, we have established local stability for integer-order and fractional-order models, and in “Global stability analysis of endemic steady state for integer order model” section, we have derived the global stability of the integer-order model, and also we have analysed the impact of the fear parameter on the infected density in “The impact of fear level k on the infected density” section. Numerical simulation and discussion to verify theoretical results, stability region, and effect of fear level on infected population are given in “Results and discussion” section. Finally, the conclusion is discussed in “Conclusion” section.

## Preliminaries

In this section, fractional-order derivative in the Caputo sense [29], equilibrium points of the system, and some vital theorem of the local stability of the fractional-order system are given.

### Definition 2.1

The Caputo-type fractional derivatives of order $\alpha >0$, for $n-1<\alpha <n$, $n\in N$ and for a function $f\in L^{1}(R^{+})$ is given by

1 $$\begin{aligned} ^{c}_{t_{0}}D^{\alpha }_{t}f(t)=\frac{1}{\Gamma (k-\alpha )}\int _{t_{0}}^t{(t-\eta )}^{(k-\alpha -1)}f^{k}(\eta )d\eta \end{aligned}$$

where the function *f*(*t*) is continuous derivative up to the order $(n-1)$ and $\Gamma (.)$ denotes the Gamma function.

Considering the following Caputo fractional-order differential equations containing n equations for $0<\alpha <1$:

2 $$\begin{aligned} ^{c}_{t_{0}}D^{\alpha }_{t}x(t)=f(t,x(t)) \end{aligned}$$

with $x(t_{0})=x_{0}$ where $f:[t_{0},\infty )\times \Omega \rightarrow R^{n}$, there exists unique solution of the fractional-order differential equation on the region $[t_{0},\infty )\times \Omega$, if *f*(*t*, *x*(*t*)) satisfies the Lipschitz condition with respect to *x*. The equilibrium points of system (2) are obtained by equating $f(t,x(t))=0$. Now, the following theorems describe the stability of the equilibrium points.

### Lemma 2.2

[30] Let us assume that $\alpha \in (0,1]$ and both the functions f(t) and its fractional derivative $^{c}_{t_{0}}D^{\alpha }_{t}x(t)$ be elements of the metric space *C*[*a*, *b*]. If $^{c}_{t_{0}}D^{\alpha }_{t}x(t)\ge 0$, then the function *f*(*t*) is a monotone increasing and the function is monotone decreasing if $^{c}_{t_{0}}D^{\alpha }_{t}x(t)\le 0$ for all $t\in [a,b]$.

### Lemma 2.3

[31] Let us consider that $x: [t_0,\infty )\rightarrow R$ be continuous function and satisfies the following: $^{c}_{t_{0}}D^{\alpha }_{t}x(t) +\mu x(t)\le \rho$, $x(t_0)=x_0$, $t_0\ge 0$, $\rho , \mu \in R$, $\mu \ne 0$ and $\alpha \in (0,1)$. Then, we have the inequality $x(t)\le (x_0-\frac{\rho }{\mu })E_\alpha [-\mu (t-t_0)^\alpha ]+\frac{\rho }{\mu }$ for all $t\ge t_0$. Here $E_\alpha$ is the Mittag-Leffler function of one parameter.

### Theorem 2.4

[32] Let the Jacobian matrix $J(x^{*})$ of system (2) is evaluated at the equilibrium point $x^{*}$. $\lambda _{i}$, where $i=1,2,3,....,n$ be the eigenvalues of $J(x^{*})$ and the equilibrium point $x^{*}$ is to be a saddle point if few of $\lambda _{i}$ satisfy $\vert {arg\lambda _{i}}\vert >\frac{\alpha \pi }{2}$ and few others satisfy $\vert {arg\lambda _{i}}\vert <\frac{\alpha \pi }{2}$.

### Theorem 2.3

[33] Considering the fractional-order differential system

(i) The necessary and sufficient condition for an equilibrium point $x^*$ is locally asymptotically stable if all eigenvalues $\lambda _i$ of $J(x^{*})$ satisfy $\vert {arg\lambda _{i}}\vert >\frac{\alpha \pi }{2}$, where $i=1,2,3,....,n$.
(ii) The necessary and sufficient condition for an equilibrium point $x^*$ is stable if all eigenvalues $\lambda _i$, where $i=1,2,3,....,n$ of $J(x^{*})$ satisfying $\vert {arg\lambda _{i}}\vert \ge \frac{\alpha \pi }{2}$ and eigenvalues with $\vert {arg\lambda _{i}}\vert =\frac{\alpha \pi }{2}$ have the same geometric and algebraic multiplicity.
(iii) The necessary and sufficient condition for an equilibrium point $x^*$ is unstable if for some eigenvalues $\lambda _i$,$i=1,2,3,....,n$ of $J(x^{*})$ which satisfy $\vert {arg\lambda _{i}}\vert <\frac{\alpha \pi }{2}$.

## Methods

### Model formulation

An SIS epidemic model of disease transmission is proposed and studied. Accordingly, the total population consists of two compartments denoting S(t) and I(t), where S(t) and I(t) represent the number of susceptible and infected population at time t, respectively. For the mathematical formulation of the model, the following hypothesizes are adopted as follows:

1. Varying population recruitment is considered, i.e. constant rate of recruitment A and a birth rate r on both susceptible and infected populations.
2. Horizontally transmitted disease among the population refers to the susceptibility of all newborn, and it always exists in society, i.e. never dies out.
3. The disease is transmitted by direct contact with the infected population and causes the susceptible population to diminish at a rate $\frac{\beta SI}{1+kI}$, where the per capita effective contact rate of obtaining the disease is represented by $\beta$.
4. Factor $\frac{1}{1+kI}$ stands for the anti-infected effect on the susceptible population where *k* is identified as the level of fear.
5. The individuals in both compartments diminish due to the natural death rate $\mu$.
6. Infected population becomes susceptible after recovery from the disease infection.
7. The infected population is reduced when the infected individuals recover at a rate $\delta$ and disease encourage the death rate *d*.

### Model formulation of integer-order system

According to the above hypothesis, the susceptible population increases due to constant recruitment, birth in both populations, and recovery from disease but decreases by natural death and contact with the infected population. The infected population increases due to contact with susceptible populations and declines by recovery, natural, and disease-related death rates. Mathematically, the system of nonlinear differential equations (3) is represented with initial conditions $S(0), I(0)>0$.

3 $$\begin{aligned}&\frac{dS}{dt}=A+rS+rI-\frac{\beta SI}{1+kI}+\delta I-\mu S \nonumber \\&\frac{dI}{dt}=\frac{\beta SI}{1+kI}-\mu I-dI-\delta I \end{aligned}$$

### Model formulation of fractional-order system

In this section, we replace the first-order differential defining in (3) by Caputo fractional-order derivatives and we construct a new model with fractional-order differential equations, which can be written as

4 $$\begin{aligned}&^{c}_{t_{0}}D^{\alpha }_{t}S(t)=A+rS+rI-\frac{\beta SI}{1+kI}+\delta I-\mu S\nonumber \\&\quad ^{c}_{t_{0}}D^{\alpha }_{t}I(t)=\frac{\beta SI}{1+kI}-\mu I-dI-\delta I \end{aligned}$$

where $0<\alpha <1$ is the order of the Caputo-type fractional derivatives with initial condition *S*(0), $I(0)>0$.

## Existence and uniqueness of solutions for integer- and fractional-order system:

### Lemma 4.1

Let us define the integer-order system of differential equations be $\frac{dX}{dt}=f(t,x)$, $t_0>0$ and $x(t_0)=x_0$ where $f:[t_0,\infty )\times R\rightarrow R^n$, $R\subset R^n$ be a function, if the Lipschitz condition is satisfied by *f*(*t*, *x*) with respect to *x*, then the above system possesses a unique solution on the interval $[t_0,\infty )\times R$.

### Proof

Now, we consider the region $[t_0, U]\times R$ to establish the existence and uniqueness criterion of the solutions of fractional-order system(3), where $R= \lbrace (x,y)\in R^2: max\lbrace \vert S\vert , \vert I\vert \rbrace \le M \rbrace$, *U* and *M* are two finite positive real numbers. Let $X=(S,I)$ and $Y=(\overline{S}, \overline{I})$ be two points in *R* and consider a mapping $E: R\rightarrow R^2$ defined by $E(X)=(E_1(x),E_2(x))$, where $E_1(X)= A+rS+rI-\frac{\beta SI}{(1+kI)}+\delta I-\mu S$ $E_2(X)=\frac{\beta SI}{(1+kI)}-(d+\mu +\delta )I$ For any *X*, *Y* $\in R$, $\Vert E(X)-E(Y) \Vert = \vert E_1(X)-E_1(Y)\vert +\vert E_2(X)-E_2(Y)\vert$ $= \vert r(S-\overline{S}+r(I-\overline{I})+ (\frac{\beta \overline{S} \overline{I}}{(1+k\overline{I})}-\frac{\beta SI}{1+kI})+\delta (I-\overline{I})-\mu (S-\overline{S})\vert +\vert \frac{\beta SI}{1+kI}-\frac{\beta \overline{S} \overline{I}}{(1+k\overline{I})}-(d+ \mu +\delta )(I-\overline{I}) \vert$ $\leqslant r\vert S-\overline{S}\vert +r\vert I-\overline{I}\vert +\beta \frac{\vert \overline{S}(\overline{I}-I)+I(\overline{S}-S)\vert }{(1+kI)(1+k\overline{I})} +\frac{kI \overline{I}\vert S- \overline{S}\vert }{(1+kI)(1+k\overline{I})} +\delta \vert I- \overline{I} \vert + \mu \vert S-\overline{S}\vert + \beta \frac{\vert I(S-\overline{S})+\overline{S}(I-\overline{I})\vert }{(1+kI)(1+k\overline{I})}+\frac{kI \overline{I}\vert S- \overline{S}\vert }{(1+kI)(1+k\overline{I})}$ $\le [r+\frac{2\beta M}{(1+k\overline{I})}+\mu +\frac{2 k M \overline{I}}{(1+k\overline{I})}](S-\overline{S}) +[\frac{2 \beta \overline{S}}{(1+k\overline{I})}+ \delta +(d+\mu +\delta )](I-\overline{I})\le L \Vert X- Y\Vert$ Where, $L=max\lbrace [r+\frac{2\beta M}{(1+k\overline{I})}+\mu +\frac{2 k M \overline{I}}{(1+k\overline{I})}], [\frac{2 \beta \overline{S}}{(1+k\overline{I})}+ \delta +(d+\mu +\delta )] \rbrace$ Thus, *E*(*X*) satisfies the Lipschitz condition, which implies the existence and uniqueness of the solution of integer-order system (3). $\square$

### Lemma 4.2

Let us assume the order of the fractional-order system of the differential equation be $\alpha \in (0,1]$ such that $^{c}_{t_{0}}D^{\alpha }_{t}x(t)=f(t,x)$, $t_0>0$ and $x(t_0)=x_0$ where, $f:[t_0,\infty )\times R\rightarrow R^n$, $R\subset R^n$ be a function, if the Lipschitz condition is satisfied by *f*(*t*, *x*) with respect to *X*, then the above system possesses a unique solution on the interval $[t_0,\infty )\times R$.

### Proof

Proof is similar to Lemma 4.1. $\square$

## Non-negativity and boundedness of solutions for integer- and fractional-order system

### Lemma 5.1

All the solutions of system (3), which initiate in $R{_{+}^2}$, are uniformly bounded.

### Proof

Let S(t) and I(t) be any solution of system (3) with nonnegative initial condition. To prove the uniform boundedness of the solution assuming the function $N(t)=S(t)+ I(t)$, $\frac{dN}{dt}+\mu N(t)$ $=\frac{dS}{dt}+\frac{dI}{dt}+\mu S(t)+\mu I(t)$ $= A+rS+rI-dI\le A+r(S+I)\le \vert A+ r(S+I)\vert \le A+ r\vert S+I \vert$ $\le A + 2Mr$, where $max\lbrace \vert S\vert , \vert I\vert \rbrace \le M \rbrace$ and then we obtain $N(t)\le \frac{A+2Mr}{\mu }(1-e^{-\mu t})+N_{0}e^{-\mu t}$ Thus, $lim _{t{\rightarrow \infty }}Sup N(t)\le \frac{A+2Mr}{\mu } \Rightarrow N(t)\le \frac{A+2Mr}{\mu }$ for all t [34]. Therefore, each solution of system (3) initiating in the region $R{_{+}^2}$is lying in the region $\Psi =\lbrace (S,I)\in R{_{+}^2} : S+I\le \frac{ A+ 2Mr}{\mu }\rbrace$. $\square$

### Lemma 5.2

Each solution of system(4) initiating in $R{_{+}^2}$ is nonnegative and uniformly bounded.

### Proof

First, we consider that $X_{t_0}=(S_{t_0},I_{t_0})\in R{_{+}^2}$ is an initial solution of system (4). Now, we establish that any solution $X(t)\in R{_{+}^2}$ is nonnegative. Assuming that T is a real number such that $t_0\le t< T$ and

$$\begin{aligned} S(t)&= 0 ; t_0 \le t \le T \\ &= < 0 ; t= T^+ \end{aligned}$$

The first equation of (4) implies that $^{c}_{t_{0}}D^{\alpha }_{t}S(t)\vert _{S(T)}=0$. Also from Lemma 2.2 implies that $S(T^+)=0$ which contradicts the assumption $S(T^+)<0$. Hence, for any $t\in [t_0, \infty )$ we obtain $S(t)\ge 0$ and similarly we can prove that $I(t)\ge 0$ for all $t\in [t_0, \infty )$.

To prove the uniform boundedness of the solutions, let us define a function $V(t)= S(t)+ I(t)$. Then, $^{c}_{t_{0}}D^{\alpha }_{t}V(t)+\mu V(t)$ $=^{c}_{t_{0}}D^{\alpha }_{t}S(t) +^{c}_{t_{0}}D^{\alpha }_{t}I(t)+\mu S(t)+ \mu I(t)$ $= A+rS+rI-dI\le A+r(S+I)\le \vert A+ r(S+I)\vert \le A+ r\vert S+I \vert$ $\le A + 2Mr$.

Therefore, Lemma 2.3 implies that $V(t)\le (V(t_0)-\frac{\lbrace A+ 2Mr\rbrace }{\mu })E_\alpha [-d(t-t_0)^\alpha ] + \frac{\lbrace A+ 2Mr\rbrace }{\mu }\rightarrow \frac{\lbrace A+ 2Mr\rbrace }{\mu }$ as $t\rightarrow \infty$. Hence, each solution of system (4) initiated in the region $R{_{+}^2}$ is lying in the region $\Psi =\lbrace (S,I)\in R{_{+}^2} : S+I\le \frac{\lbrace A+ 2Mr\rbrace }{\mu }\rbrace$. $\square$

## Existence of equilibrium points for the integer-order model

In this section, we have discussed the existence of the equilibrium points of system (3), and the value of $S^{*}$ is obtained by equating the second equation of system (3) to zero, we have $S^{*}=g(1+kI^{*})$. Now incorporating the expression $S^{*}$ in the first equation of model (3) is equating to zero and solving we get the following quadratic equation in terms of $I^{*}$,

5 $$\begin{aligned} A_{2}(I^{*})^2+A_{1}(I^{*})+A_{0}=0 \end{aligned}$$

where $A_{2}=k[kg(r-\mu )+(r+\delta )-g\beta ]$ $A_{1}=[Ak+2kg(r-\mu )+(r+\delta -g\beta )]$ $A_{0}=A+g(r-\mu )$ We analyse the model (3) under two cases when the natural birth rate is less or greater than the natural death rate, i.e. $r<\mu$ and $r>\mu$ .

### Case I $(r<\mu )$

#### Theorem 6.1

- The model (3) has a unique disease-free equilibrium point $E_{0}(\frac{A}{\mu -r},0)$
- A unique endemic equilibrium point $E_{1}(S_1,I_1)$, where $I_1=\frac{A-g(\mu -r)}{d+(\mu -r)(1+gk)}$; $S_1=g(1+kI^{*})$ exists if *r* lies between two threshold values $\mu -\frac{A}{g}<r<\mu,$ i.e. $A_{2}<0$, $A_{1}<0$ and $A_{0}>0$.
- No endemic equilibrium point exists if $r<\mu -\frac{A}{g},$ i.e. $A_{2}<0$, $A_{1}<0$ and $A_{0}<0$ under the condition.

#### Proof

It will be proved by using Descartes’s rule of sign. $\square$

### Case II $(r>\mu )$

#### Theorem 6.2

- A unique endemic equilibrium point $E_{2}(S_2,I_2)$, $I_2=\frac{A+g(r-\mu )}{d-(r-\mu )(1+gk)}$ and $S_2=g(1+kI^{*})$ exists if r lies between two threshold values $\mu +\frac{d-kA}{1+2kg}<r<\mu +\frac{d}{1+gk}$, when $d>kA$ that is if $A_{2}<0$, $A_{1}>0$ and $A_{0}>0$
- No endemic equilibrium point exists if $\mu +\frac{d}{1+gk}<r,$ i.e. $A_{2}>0$, $A_{1}>0$ and $A_{0}>0$ under the condition.

#### Proof

It will be proved by using Descartes’s rule of sign. $\square$

From this discussion, it is observed that when $r<\mu$ there exist two equilibrium points, disease free $E_{0}(S_0,I_0)$ and the endemic equilibrium point $E_{1}(S_1,I_1)$, and when $r>\mu$ only endemic equilibrium point $E_{2}(S_2,I_2)$ exists.

Now, we define the basic reproduction number $R_{0}=\frac{A\beta }{(\mu -r)(d+\delta +\mu )}$; it represents the average number of secondary infections that occur when one infectious individual is entered into a completely susceptible population.

## Existence of the equilibrium points for fractional-order model

The equilibrium points of the fractional-order model defining on (4) are obtained by solving the equation $^{c}_{t_{0}}D^{\alpha }_{t}S(t)=0$ and $^{c}_{t_{0}}D^{\alpha }_{t}I(t)=0$, i.e.

$$\begin{aligned}&A+rS+rI-\frac{\beta SI}{1+kI}+\delta I-\mu S=0\\&\frac{\beta SI}{1+kI}-\mu I-dI-\delta I=0 \end{aligned}$$

We get two equilibrium points same as the equilibrium points obtained in the integer-order system of differential equation defining in “Existence of equilibrium points for the integer-order model” section.

## Local stability analysis at different equilibrium points of integer-order system model

### Case I $(r<\mu )$

#### Theorem 8.1

In the absence of disease (I=0), the equilibrium point $E_{0}$ of the model (3) is locally asymptotically stable if $R_{0}<1$ and unstable if $R_{0}>1$.

#### Proof

The stability of the disease-free equilibrium point is examined by using the Jacobian matrix for model (3) at $E_{0}$

6 $$\begin{aligned} J(E_{0})=\begin{bmatrix} r-\mu &{}r-\frac{\beta A}{\mu -r}+\delta \\ 0 &{}\frac{\beta A}{\mu -r}-(d+\mu +\delta )\\ \end{bmatrix} \end{aligned}$$

Thus, the corresponding two eigenvalues of $J(E_{0})$ are $\lambda _{1}=-(\mu -r)$ and $\lambda _{2}= [\frac{\beta A}{(\mu -r)}-(d+\mu +\delta )]$ in which $-(\mu -r)$ is always negative. Another eigenvalue is represented as $(d+\mu +\delta )(R_{0}-1)<0$ which is to be negative if $R_{0}< 1$, i.e. $E_{0}$ is locally asymptotically stable if $R_{0}< 1$, and $\lambda _{2}$ is positive if $R_{0}> 1$ indicates that $E_{0}$ is unstable. Thus, disease-free steady state changes its stability when $R_{0}$ changes through critical value $R_{0}=1$. So $E_{0}$ admits bifurcation, and $R_{0}$ represents the threshold quantity shown in Fig. 2. $\square$

#### Theorem 8.2

The dynamical behaviour of system (3) undergoes a transcritical bifurcation with critical value $R_{0}=1$.

#### Proof

In this section, we analyse the behaviour of system (3) when the basic reproduction number is equal to one. We notice that the Jacobian matrix of system (3) evaluated at $R_{0}=1$ and $\beta =\beta ^{*}=\frac{(d+\mu +\delta )(\mu -r)}{A}$ has a simple zero eigenvalue and another eigenvalue with a negative real part. Therefore, the stability behaviour of equilibrium points at $R_{0}=1$ cannot be determined using linearization. So we apply the central manifold theorem of system (3) with the following assumptions: $S=x_{1}$ and $I=x_{2}$, and then the system can be written as

7 $$\begin{aligned}&\frac{dx_{1}}{dt}=A+rx_{1}+rx_{2}-\frac{\beta x_{1} x_{2}}{1+kx_{2}}+\delta x_{2}-\mu x_{1}=f_{1}(x)\nonumber \\&\frac{dx_{2}}{dt}=\frac{\beta x_{1}x_{2}}{1+kx_{2}}-\mu x_{2}-dx_{2}-\delta x_{2}=f_{2}(x) \end{aligned}$$

Now, the Jacobian matrix at $R_{0}=1$ and $\beta =\beta ^{*}$ will be obtained as

$$\begin{aligned} J=\begin{bmatrix} r-\mu &{} r-\frac{A\beta ^{*}}{\mu -r}+\delta \\ 0 &{} \frac{A\beta ^{*}}{\mu -r}-(d+\mu +\delta )\\ \end{bmatrix} \end{aligned}$$

Let $w=[w_{1},w_{2}]$ and $u=[u_{1},u_{2}]^{T}$ be the left and right eigenvectors of J corresponding to zero eigenvalue.

Then, we have $w_{1}=0$, $w_{2}= 0$, $u_{1}=\frac{-(d+\mu -r)}{\mu -r}$, $u_{2}=1$. The nonzero partial derivatives is associated with the function of system (7) is evaluated at $R_{0}=1$ and $\beta =\beta ^{*}$ are $(\frac{\partial ^{2}f_{2}}{\partial x_{1} \partial x_{2}})_{E_{0}}=\frac{\beta ^{*}}{(1+kI_{0})^{2}}$; $(\frac{\partial ^{2}f_{2}}{\partial x_{2}^{2}})_{E_{0}}=\frac{-2\beta ^{*}Ak}{\mu -r}$ ; $(\frac{\partial ^{2}f_{2}}{\partial x_{2} \partial \beta ^{*}})_{E_{0}} =\frac{A}{\mu -r}$ Now, we proceed in obtaining the associated bifurcation coefficients, respectively, denoted by $a_{1}$ and $b_{1}$ being described in theorem 4.1 of Castillo-chavez and Song [35].

$$\begin{aligned}&a_{1}=\sum _{k,i,j=1}^{2}w_{k}u_{i}u_{j}(\frac{\partial ^{2}f_{k}}{\partial x_{i} \partial x_{j}})_{E_{0}} =w_{2}[u_{1}u_{2}(\frac{\partial ^{2}f_{2}}{\partial x_{1} \partial x_{2}})_{E_{0}}] =\frac{-(d+\mu -r)(d+\mu +\delta )}{A}<0\\&b_{1}=\sum _{k,i=1}^{2}w_{k}u_{i}(\frac{\partial ^{2}f_{k}}{\partial x_{i} \partial \beta ^{*}})_{E_{0}}=w_{2}[u_{2}(\frac{\partial ^{2}f_{2}}{\partial x_{2} \partial \beta ^{*}})_{E_{0}}]=\frac{A}{\mu -r}>0 \end{aligned}$$

Thus, theorem 4.1 of Castillo-Chavez indicates that if the bifurcation coefficients $a_{1}$ give the negative value and $b_{1}$ gives the positive value, we conclude the following result that DFE changes its stability from stable to unstable at $R_{0}=1$, and a positive equilibrium point exists $R_{0}$ crosses one. Hence, system (3) undergoes a transcritical bifurcation at $R_{0}=1$. $\square$

#### Theorem 8.3

The endemic equilibrium point $E_{1}(S_1,I_1)$ of the model (3) is always asymptotically stable for $R_{0}>1$ when it exists.

#### Proof

An endemic equilibrium point $E_{1}(S_1,I_1)$ of the model (3) is the steady state where the disease may persist in the population. It suggests that all the infected compartments are not empty, whereas positive endemic equilibrium point $E_1(S_1,I_1)$ of the model (3) exists if $A-(\mu -r)g>0$ which indicates that $\frac{A}{(\mu -r)g}>1,$ i.e. $R_{0}>1.$ The variational matrix for system (3) at $E_1(S_1,I_1)$ is

8 $$\begin{aligned} J(E_1(S_1,I_1))=\begin{bmatrix} r-\mu -\frac{\beta I_1}{1+kI_1} &{} r-\frac{\beta S_1}{(1+kI_1)^{2}}+\delta \\ \frac{\beta I_1}{1+kI_1} &{} \frac{\beta S_1}{(1+kI_1)^{2}}-(d+\mu +\delta )\\ \end{bmatrix} \end{aligned}$$

Now, the trace of $J(E_{1})$ is

$$\begin{aligned} tr J(E_{1})=-(\mu -r)-\frac{\beta I_1(1+gk)}{1+kI_1}<0 \end{aligned}$$

and

$$\begin{aligned} det J(E_{1})=[(\mu -r)(1+gk)+d](\frac{\beta I_1}{(1+kI_1)}>0 \end{aligned}$$

Thus, it is always true $tr J(E_{1})<0$ and $det J(E_{1})>0$. Applying the Routh–Hurwitz criteria, it follows that the eigenvalues of the above variational matrix have negative real parts, which implies that the endemic equilibrium $E_{1}(S_1,I_1)$ is always locally asymptotically stable when it exists. $\square$

### Case II $(r>\mu )$

#### Theorem 8.4

The endemic equilibrium point $E_{2}(S_2,I_2)$ is asymptotically stable when r lies between two threshold values $\mu +\frac{d-kA}{1+2kg}$ and $\mu +\frac{A\beta (1+gk)}{(kA+d)}$ and unstable if $\mu +\frac{A\beta (1+gk)}{(kA+d)}<r<\mu +\frac{d}{1+gk}$.

#### Proof

Another endemic equilibrium point $E_{2}(S_2,I_2)$ exists when the growth rate *r* is greater than the natural death rate $\mu$, whereas the existence of positive $E_{2}(S_2,I_2)$ occurs when r lies between two threshold values $\mu +\frac{d-kA}{1+2kg}<r<\mu +\frac{d}{1+gk}$ whenever $d>kA$. The trace of $J(E_{2})$ is

9 $$\begin{aligned}&tr J(E_{2})=r-\mu -\frac{\beta I_2}{(1+kI_2)}+\frac{\beta S_2}{(1+kI_2)^2}-(d+\mu +\delta )\nonumber \\&\quad =r-\mu -\frac{\beta I_2}{(1+kI_2)}-g\beta \frac{kI_2}{(1+kI_2)}\nonumber \\&\quad =r-\mu -(1+gk)\frac{\beta I_2}{(1+kI_2)} \end{aligned}$$

So $tr J(E_{2})<0$ holds whenever $r-\mu <(1+gk)\frac{\beta I_2}{(1+kI_2)}$ and after simplification,

$$\begin{aligned} r<\mu +\frac{A\beta (1+gk)}{(kA+d)} \end{aligned}$$

and $tr J(E_{2})>0$ if the condition $\mu +\frac{A\beta (1+gk)}{(kA+d)}<r$ hold. And also

10 $$\begin{aligned}&detJ(E_{2})=\frac{\beta I_2}{(1+kI_2)}[-(r-\mu )gk+g\beta -(r+\delta )]\nonumber \\&\quad =\frac{\beta I_2}{(1+kI_2)}[d-(r-\mu )(1+gk)]>0 \end{aligned}$$

Thus, $detJ(E_{2})$ is always positive but $tr J(E_{2})$ is negative when $r<\mu +\frac{A\beta (1+gk)}{(kA+d)}$. So by Routh–Hurwitz criteria the eigenvalues have negative real part, implying that $E_{2}$ is asymptotically stable when $\mu +\frac{d-kA}{1+2kg}<r<min(\mu +\frac{d}{1+gk},\mu +\frac{A\beta (1+gk)}{(kA+d)} )$, i.e. $\mu +\frac{d-kA}{1+2kg}<r<\mu +\frac{A\beta (1+gk)}{(kA+d)})$ and $E_{2}$ is unstable if $\mu +\frac{A\beta (1+gk)}{(kA+d)}<r<\mu +\frac{d}{1+gk}$ condition hold. $\square$

## Local stability analysis at different equilibrium points for fractional-order model

Now, the local stability analysis of the equilibrium points is to be observed using the standard linearization technique.

### Case I $(r<\mu )$

#### Theorem 9.1

Stability analysis of disease-free equilibrium point $E_{0}(\frac{A}{\mu -r},0)$ for the fractional model.

#### Proof

The Jacobian matrix of the model (4) is evaluated at the equilibrium point $E_{0}$ is already defined in (4.1) and the eigenvalues of $J(\frac{A}{(\mu -r)},0)$ are $\lambda _{1}=(r-\mu )<0$ and $\lambda _{2}=\frac{\beta A}{\mu -r}-(d+\mu +\delta )$. So we have $\vert {arg\lambda _{1}}\vert =\vert {arg\lambda _{2}}\vert =\pi$ and which satisfies the condition $\vert {arg\lambda _{i}}\vert >\frac{\alpha \pi }{2}$. Hence, disease-free equilibrium point$E_{0}$ is locally asymptotically stable. $\lambda _{2}$ is positive and $\lambda _{1}$ is negative, which implies that $\vert {arg\lambda _{2}}\vert =0$ and $\vert {arg\lambda _{1}}\vert =\pi$. Hence, DFE $E_{0}$ is a saddle-node. $\square$

#### Theorem 9.2

Stability analysis at the endemic equilibrium point $E_{1}(S_1,I_1)$ of the fractional-order model.

#### Proof

The Jacobian matrix for the fractional-order model at the endemic steady state $E_1(S_1,I_1)$ is already defined in (8). The trace of $J(E_{1})$ is

11 $$\begin{aligned}&tr J(E_{1})=-(\mu -r)-(1+gk)\frac{\beta I_1}{(1+kI_1)}<0\end{aligned}$$

12 $$\begin{aligned}&\quad detJ(E_{1})=\frac{\beta I_1}{(1+kI_1)}[d+(\mu -r)(1+gk)]>0 \end{aligned}$$

In general, the eigenvalues of $J(S_1,I_1)$ are given by

13 $$\begin{aligned}&\lambda _{1}=\frac{1}{2}[ tr(J(S_1,I_1))+(tr^{2}(J(S_1,I_1))-4det(J(S_1,I_1)))^\frac{1}{2}]\nonumber \\&\quad \lambda _{2}=\frac{1}{2}[ tr(J(S_1,I_1))-(tr^{2}(J(S_1,I_1))-4det(J(S_1,I_1)))^\frac{1}{2}] \end{aligned}$$

Since $tr(J(E_{1}))$ is always negative under this case, the proof will be divided into two cases.

*Case*(*a*) When $tr(J(E_{1})) <0$ and $(tr^{2}(J(E_{1}))-4det(J(E_{1}))\ge 0$, then from equation (9.3) represents, both eigenvalues $\lambda _{1}$ and $\lambda _{2}$ of $(J(E_{1}))$ are negative, which implies that $\vert {arg\lambda _{1}}\vert >\frac{\alpha \pi }{2}$ and $\vert {arg\lambda _{2}}\vert >\frac{\alpha \pi }{2}$. Hence, according to theorem 2.5, the equilibrium point $E_{1}$ is locally asymptotically stable.

*Case*(*b*) When $tr(J(E_{1}))<0$ and $(tr^{2}(J(E_{1}))-4det(J(E_{1}))<0$, then from equation (9.3), we obtain a pair of complex conjugate eigenvalues $\lambda _{1}$ and $\lambda _{2}=\overline{\lambda _{1}}$. Since $tr(J(E_1(S_{1},I_{1})))<0$, so both the eigenvalues have negative real part, which implies that $\vert {arg\lambda _{1}}\vert >\frac{\alpha \pi }{2}$ and $\vert {arg\lambda _{2}}\vert >\frac{\alpha \pi }{2}$. Hence, according to theorem 2.5, the equilibrium point $E_{1}$ is locally asymptotically stable. $\square$

### Case II $(r>\mu )$

#### Theorem 9.3

Analysis at the endemic equilibrium point $E_{2}(S_2,I_2)$ for the fractional-order model.

#### Proof

The trace and determinant of the Jacobian matrix $J(E_{2})$ are given below. The trace of $J(E_{2})$ is

$$\begin{aligned}&tr J(E_{2})=r-\mu -(1+gk)\frac{\beta I_2}{(1+kI_2)}\\&\quad detJ(E_{2}) =\frac{\beta I_2}{(1+kI_2)}[d-(r-\mu )(1+gk)]>0 \end{aligned}$$

Whenever the positive endemic equilibrium point $E_{2}$ exists under the condition defined in Th 6.2(a)

(*i*) When $tr(J(E_{2}))\le 0$ three cases will arise.

*Case*(*a*) :  If $tr(J(E_{2}))= 0$, then we obtain a pair of complex conjugate eigenvalues $\lambda _{1}$ and $\lambda _{2}=\overline{\lambda _{1}}$ from equation (7.3). Since $Re(\lambda _{1})=Re(\lambda _{2})=0$, so we have $\vert {arg\lambda _{1}}\vert =\frac{ \pi }{2}$ and $\vert {arg\lambda _{2}}\vert =\frac{\pi }{2}$ which satisfies the stability condition. Hence, the endemic equilibrium point $E_{2}$ is asymptotically stable.

*Case*(*b*) :  If $tr(J(E_{2}))< 0$ and $(tr^{2}(J(E_{2}))-4det(J(E_{2})\ge 0,$ then both the eigenvalues of $J(E_{2})$ are negative, which satisfies the condition $\vert {arg\lambda _{1}}\vert >\frac{\alpha \pi }{2}$ and $\vert {arg\lambda _{2}}\vert >\frac{\alpha \pi }{2}$. So, according to theorem 2.5, equilibrium point $E_{2}$ is locally asymptotically stable.

*Case*(*c*):  If $tr(J(E_{2}))< 0$ and $(tr^{2}(J(E_{2}))-4det(J(E_{2}))< 0$, then we obtain a pair of complex conjugate eigenvalues $\lambda _{1}$ and $\lambda _{2}=\overline{\lambda _{1}}$ Since $tr(J(E_{2}))< 0$, both the eigenvalues have negative real part, which implies that $\vert {arg\lambda _{1}}\vert >\frac{\alpha \pi }{2}$ and $\vert {arg\lambda _{2}}\vert >\frac{\alpha \pi }{2}$ which satisfies the asymptotically stable condition. Therefore, $E_{2}$ is asymptotically stable.

So *Case*(*a*), *Case*(*b*) and *Case*(*c*) implies that the equilibrium point $E_{2}$ is locally asymptotically stable if $tr(J(E_{2}))\le 0$

(*ii*) Let us assume that $tr(J(E_{2}))> 0$, $(tr^{2}(J(E_{2}))-4det(J(E_{2}))< 0$ and $\vert tr^{2}(J(E_{2}))-4det(J(E_{2}))\vert ^\frac{1}{2}>tr(J(E_{2}))tan(\frac{\alpha \pi }{2})$. So from equation (9.3), we have a pair of complex conjugate eigenvalues $\lambda _{1}$ and $\lambda _{2}=\overline{\lambda _{1}}$ such that $Im(\lambda _{1})=- Im(\lambda _{2})=[4det(J(E_{2}))-tr^{2}(J(E_{2}))]^\frac{1}{2}>0$ and $Re(\lambda _{1})=Re(\lambda _{2})=tr(J(E_{2}))> 0$. From our assumptions, we obtain $Im(\lambda _{1})>Re(\lambda _{1})tan(\frac{\alpha \pi }{2})$ and $- Im(\lambda _{2})>tr(J(E_{2}))tan(\frac{\alpha \pi }{2})$ which implies that $\vert {arg\lambda _{i}}\vert >\frac{\alpha \pi }{2}$. So, according to theorem 2.5, equilibrium point $E_{2}$ is asymptotically stable.

(*iii*) If $tr^{2}(J(E_{2}))-4det(J(E_{2}))<0$, $tr^{2}(J(E_{2}))>0$ and $\vert tr^{2}(J(E_{2}))-4det(J(E_{2}))\vert ^\frac{1}{2}<tr(J(E_{2}))tan(\frac{\alpha \pi }{2})$ then $E_{2}$ is unstable. $\square$

## Global stability analysis of endemic steady state for integer-order model

### Dulac’s criteria

Now, we prove the global stability of endemic steady $E(S^{*},I^{*})$ whenever it exists, using Dulac criteria and the Poincare–Bendixson theorem. Denote the right-hand side of (1) by P(S,I),Q(S,I), and construct a Dulac’s function $\phi =\frac{(1+kI)}{\beta SI}$ where $S>0,I>0$. Then, we have

14 $$\begin{aligned}&\Delta (S,I)=\frac{\partial (\phi P)}{\partial S}+\frac{\partial (\phi Q)}{\partial I}\nonumber \\&\Delta (S,I)=\frac{-A(1+kI)}{\beta S^{2}I}-\frac{(r+\delta )(1+kI)}{\beta S^{2}}-\frac{(d+\mu +\delta )}{\beta S}<0 \end{aligned}$$

So, $\Delta (S,I)$ does not change sign and is not identically zero in the region $R^2$. Then, according to the Bendixson–Dulac criterion, there is no periodic solution in $R^2$. Now, since all solutions of system (3) are bounded and unique positive equilibrium point in the region $R^2$, by using the Poincare–Bendixson theorem $E(S^{*},I^{*})$ is globally asymptotically stable.

Taking another Dulac function, $\phi (S,I)=\frac{1}{\beta SI}$. Then we have

15 $$\begin{aligned}&\Delta (S,I)=\frac{\partial (\phi P)}{\partial S}+\frac{\partial (\phi Q)}{\partial I}\nonumber \\&\quad \Delta (S,I)=-\frac{A}{kS^{2}I}-\frac{(r+\delta )}{\beta S^{2}}-\frac{1}{(1+kI)^{2}}<0 \end{aligned}$$

We have observed that system (3) has no periodic orbit in the interior of first quadrant and only one endemic equilibrium point exists under different conditions and discussion. Hence, the endemic equilibrium point $E(S^{*},I^{*})$ is globally stable in the region $R^2$.

## The impact of fear level k on the infected density

### Case I $(r<\mu )$

To discuss the impact of the level of fear on the infected density by computing the derivative along the equilibrium value of the infected density $(I_1)$ with respect to *k* that is $\frac{dI_1}{dk}=-\frac{[A-(\mu -r)g][g(\mu -r)]}{[d+(\mu -r)(1+gk)]^2}<0$ and $\lim _{k\rightarrow \infty }\frac{dI_1}{dk}=0$, which implies that $(I_1)$ is a decreasing function of *k*, i.e. increasing the level of fear *k* can decrease the infected density but for a larger value of *k* the infected density maintains a constant level, and for these system (3) always is asymptotically stable about the equilibrium point $E_{1}$ when exists by theorem 8.3.

### Case II $(r>\mu )$

Now compute the derivative along the equilibrium value of the infected density $(I_2)$ with respect to *k* for discussing the impact of the level of fear on the infected population density that is $\frac{dI_2}{dk}=\frac{[A+(r-\mu )g][g(r-\mu )]}{[d-(r-\mu )(1+gk)]^2}>0$, $\lim _{k\rightarrow \infty }\frac{dI_2}{dk}=0$ which shows that $(I_2)$ is an increasing function of *k*, i.e. increasing the level of fear on the infected density, but for a large value of *k*, the infected density maintains a constant level, and for this system (3) is asymptotically stable about the equilibrium $E_{2}$ when exists by theorem 8.4.

## Results and discussion

The systems (3) and (4) are numerically simulated for various set parameters numerically by the fourth-order Runge–Kutta method for solving the system of ODE equations. We have used the ODE45 solver for system (3) and the FDE12 solver for system (4) and MATLAB software. This study demonstrates the stability and instability of the populations, bifurcation, and compares the stability region in both systems and specifies the value of parameters.

**Case I** $$(\mu >r)$$

Consider the data set I: $A=0.06 , r=0.035, \mu =0.04, \beta =0.025, \delta =0.03, d=0.3, k=0.01$.

**Fig. 1:**
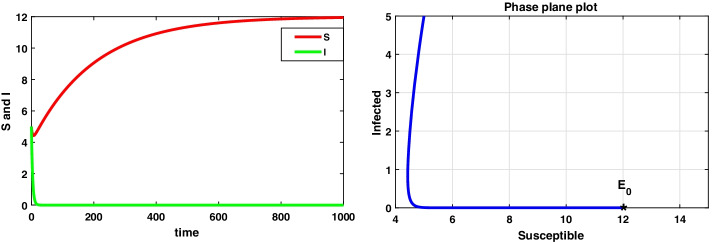
A dynamical behaviour of the integer-order model with $r<\mu$ and the corresponding two-dimensional phase plane portrait of S,I

**Fig. 2:**
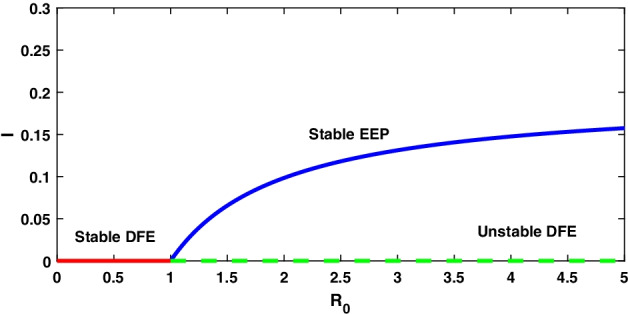
A forward bifurcation diagram of $R_0$ with the change of parameter $\beta$ on the infected population for the integer-order model with $r<\mu$ is shown

**Fig. 3:**
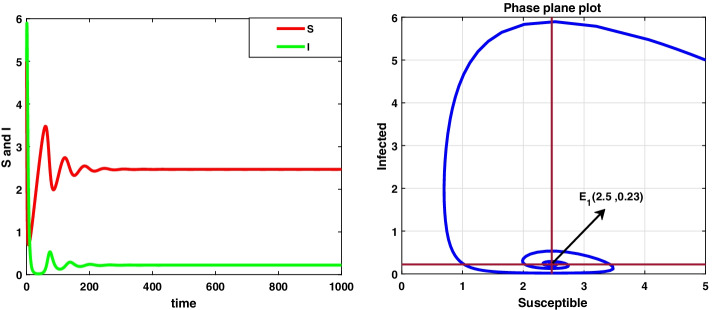
A dynamic behaviour of system (3.1) with $r<\mu$ and the corresponding two-dimensional phase portrait of S,I with susceptible and infected isocline about endemic equilibrium point $E_{1}$

For the data set I, system (3) admits a disease-free equilibrium point $E_0(14.8 ,0)$ and is asymptotically stable as the value of $R_0=0.8<1$ according to theorem 8.1, and this is displayed in Fig. 1. But when the value of infection rate increases, i.e. $\beta =0.15$, the value of $R_0=4.8>1$ and hence $E_0$ becomes saddle-node so the system is unstable. Now, the system 3.1 undergoes a forward bifurcation by theorem 8.2, and the dynamical behaviour is depicted in Fig. 2. When $R_0>1$, system (3) admits an endemic equilibrium point $E_1$ and is always asymptotically stable by theorem 8.3, and the dynamical behaviour of the system is shown in Fig. 3.

In this case, we have also observed the dynamical behaviour of system (4) is same as system (3).

**Fig. 4:**
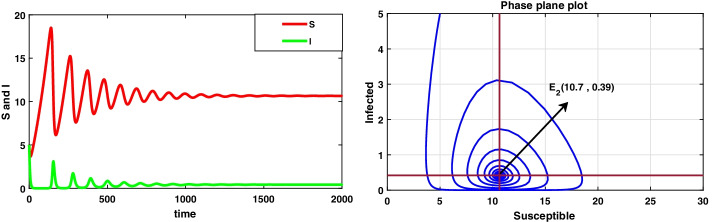
A dynamic behaviour of system (3) with $r>\mu$ and the corresponding two-dimensional phase portrait of S,I with susceptible and infected isocline about endemic equilibrium point $E_{2}$

**Case II **$(\mu <r)$ Consider the data set II: $A=0.06 , r=0.023, \mu =0.018, \beta =0.03, \delta =0.0015, d=0.3, k=0.01$ System (3) admits only the equilibrium point $E_2(10.7, 0.39)$ under the condition specified in theorem 6.2(a), i.e. when $\mu \in (0.265, 0.289)$ and system (3) is asymptotically stable under the condition mentioned in theorem 8.4, i.e. $\mu \in (0.0246, 0.289)$ and the dynamical behaviour of (3) is exhibited in Fig. 4.

**Fig. 5:**
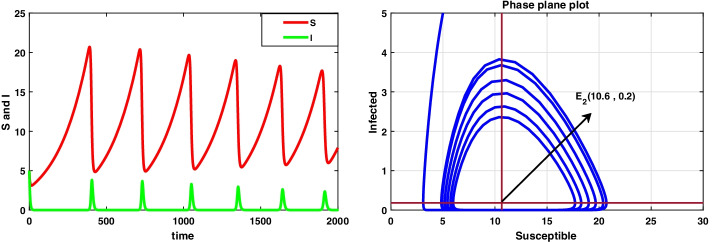
A dynamical behaviour of system (3) with $r>\mu$ and the corresponding two-dimensional phase portrait of S,I with susceptible and infected isocline about endemic equilibrium point $E_{2}$ is displayed

**Fig. 6:**
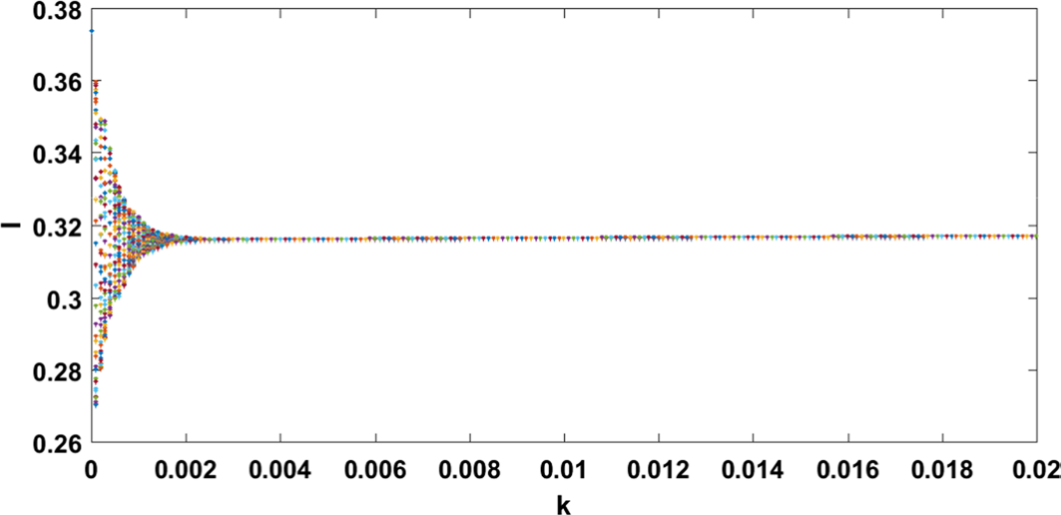
A bifurcation diagram for the infected population (I) with respect to the parameter *k*

**Fig. 7:**
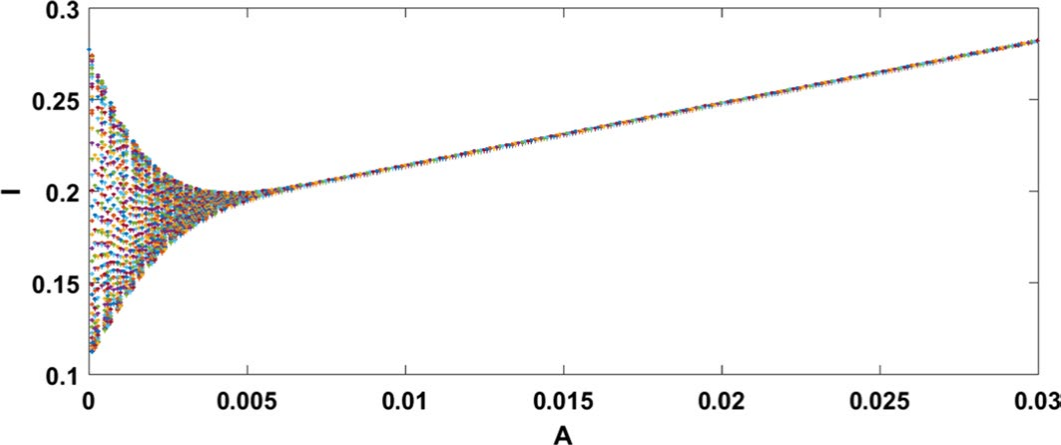
A bifurcation diagram for the infected population (I) with respect to the parameter A

**Fig. 8:**
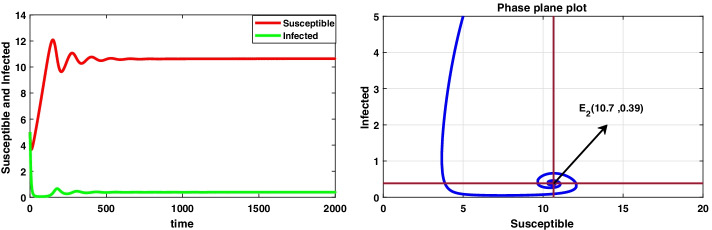
A dynamical behaviour of system (4) with $r>\mu$ and the corresponding two-dimensional phase portrait of S,I with susceptible and infected isocline about endemic equilibrium point $E_{2}$ is displayed

**Fig. 9:**
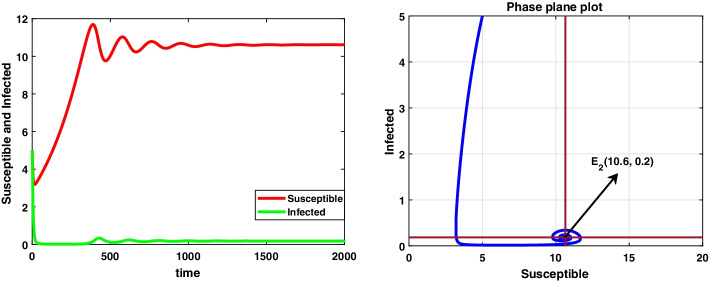
A dynamical behaviour of system (4) with $r>\mu$ and the corresponding two-dimensional phase portrait of S,I with susceptible and infected isocline about endemic equilibrium point $E_{2}$

**Fig. 10:**
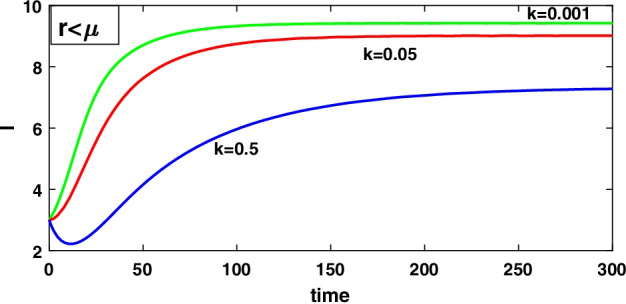
A dynamical behaviour of the infected population with different values of *k* when $r<\mu$

**Fig. 11:**
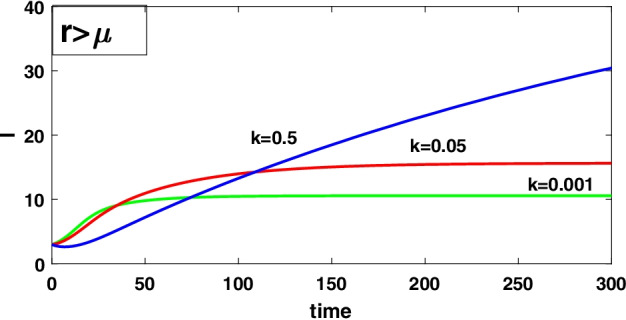
A dynamical behaviour of the infected population with different values of *k* when $r>\mu$

It has been observed that for the lower value of constant recruitment $(A= 0.001)$ and fear parameter $(k=0.001)$, system (3) admits an unstable behaviour and dynamical behaviour is displayed in Fig. 5. Hence, the system undergoes a complex dynamical system, i.e. bifurcation occurs with respect to both the parameters *A* and *k*, and the bifurcation diagram is exhibited in Figs. 6 and 7. From the figures, it has been observed that system (3) becomes unstable to stable after passing the critical value of $A= 0.007$ and $k=0.0022$. Theoretically, we have observed that system (4) has the same stability condition as system (3) as shown in Fig. 8, but from theorem 9.3(c), we can conclude that under this condition system (3) is unstable, besides system (4) has stable behaviour until the condition holds. So numerically we have discussed this phenomenon with the same data set II value with $\alpha =0.95$. Here we have observed that when system (3) is unstable in Fig. 5, then for the same parameter values, system (4) is stable, and the dynamical behaviour has displayed in Fig. 9. According to the discussion in "The impact of fear level k on the infected density" in section, we graphically represent the infected population density for different values of *k* in both cases, as depicted in Figs. 10 and 11, respectively.

According to our exploration, we have compared the integer- and fractional-order model by constructing stable and unstable regions within $\mu -r$ plane.

**Fig. 12:**
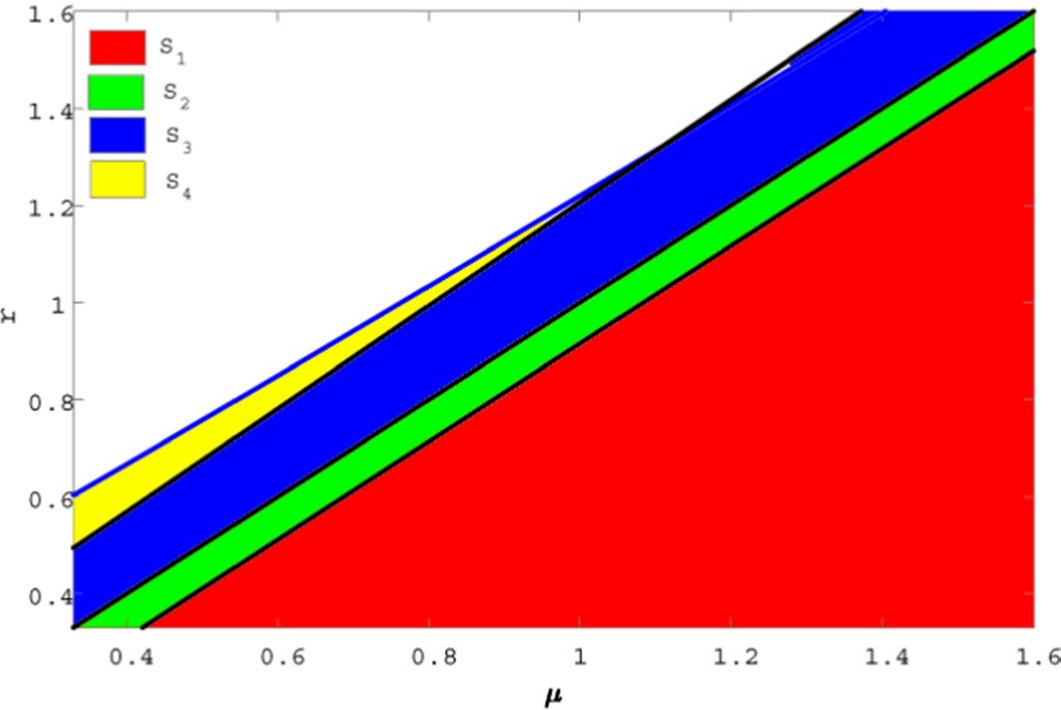
Existence of stable–unstable region of the integer-order model

**Fig. 13:**
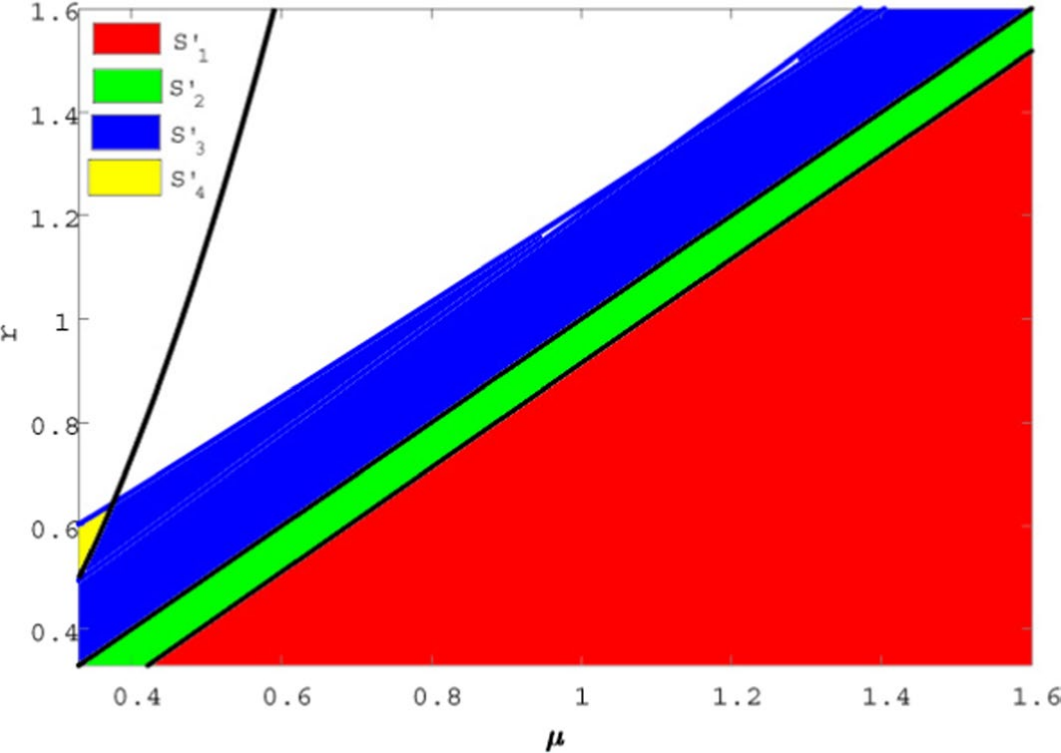
Existence of stable–unstable region for the fractional-order model

For data set III: $A=0.6 , \beta =0.08, \delta =0.005, d=0.37, k=0.04$. So for the integer-order system (3) the stability and instability regions are depicted in Fig. 12(*i*) $S_{1}=\lbrace (\mu ,r):r<\mu -\frac{A}{g}\rbrace$ represents a stable region of disease-free equilibrium point $E_{0}$ of system (3) which satisfies the stability condition of theorem 8.1 (*ii*) $S_{2}=\lbrace (\mu ,r): \mu -\frac{A}{g}<r<\mu \rbrace$ is a stable region for the endemic equilibrium point $E_{1}$ which satisfies the stability condition for $r<\mu$ in theorem 8.3 (*iii*) $S_{3}=\lbrace (\mu ,r): \mu<r<\mu +\frac{A\beta (1+gk)}{kA+d}\rbrace$ is a stable region of $E_{2}$ which satisfies the condition for $\mu <r$ in theorem 8.4. (*iv*) $S_{4}$=$\lbrace (\mu ,r):\mu +\frac{A\beta (1+gk)}{kA+d}<r<\mu +\frac{d}{(1+gk)}\rbrace$ is a unstable region for $E_{2}$ of system (3) for $\mu <r$ in theorem 8.4.Taking the value of $\alpha =0.95$ for the fractional-order system(4) exhibits the stable and unstable region in Fig. 13, where (*v*) the region $S_{1}'=\lbrace (\mu ,r):r<\mu -\frac{A}{g}\rbrace$ satisfies theorem 9.1 (*vi*) the region $S_{2}'=\lbrace (\mu ,r): \mu -\frac{A}{g}<r<\mu \rbrace$ satisfies theorem 9.2(a,b) (*vii*) the region $S_{3}'=\lbrace (\mu ,r): \mu<r<\mu +\frac{A\beta (1+gk)}{kA+d}\rbrace$ satisfies theorem 9.3 (*i*) , (*ii*) (*viii*) the region $S_{4}'$=$\lbrace (\mu ,r):\mu +\frac{A\beta (1+gk)}{kA+d}<r<\mu +\frac{d}{(1+gk)}\rbrace$ satisfies theorem 9.3 (*iii*). From Figs. 12 and 13, it is observed when $r<\mu$ the equilibrium points $E_{0}(S^{*},I^{*})$ and $E_{1}{(S^{*},I^{*})}$ are asymptotically stable in $S_{1}$, $S_{1}'$ and $S_{2}$, $S_{2}'$, respectively, and when $r>\mu$ then $E_{2}{(S^{*},I^{*})}$ is asymptotically stable in $S_{3}$ , $S_{3}'$ but unstable in $S_{4}$, $S_{4}'$ and also observed that $S_{1}$, $S_{1}'$ and $S_{2}$, $S_{2}'$ are same but $S_{3} < S_{3}'$ and $S_{4} > S_{4}'$.

## Conclusion

In this paper, we have investigated the dynamics of an integer-order and fractional-order SIS epidemic model with birth in susceptible and infected populations, constant recruitment, fear effect due to infectious disease, and compared the stability in integer-order and fractional-order systems. In our model, we have incorporated constant immigration and birth in susceptible and infected populations and studied the fear level effect on the infected population in both cases. The existence, uniqueness, non-negativity, and boundedness of the solutions for both proposed models have been discussed. We have established the existence of various equilibrium points and derived the sufficient conditions that ensure the local stability under both cases (i)$r<\mu$ and (ii) $r>\mu$. Global stability has been vindicated by using Dulac–Bendixson criterion in the integer-order model. In case I, it has been observed that there exist two equilibrium points: one is disease free which is asymptotically stable when $R_{0} < 1$ and unstable when $R_{0} > 1$. So, disease-free equilibrium point $E_{0}$ undergoes a forward transcritical bifurcation at $R_{0}=1$ with respect to the parameter $\beta$ with critical value $\beta ^{*}=\frac{(d+\delta +\mu )(\mu -r)}{A}$, hence asymptotically stable, and another one is endemic which is always asymptotically stable when $R_{0} > 1$ exists. In case II, there exists only one endemic equilibrium point, which is stable under the condition of Theorem 8.4.

By investigating the effect of the fear level on infected density, it is observed that infected density decreases in case I and increases in case II due to the increment of the fear level(*k*). In both cases, for a larger value of *k*, the infected density tends to a constant value. But in case II, for a lower value of *k* and recruitment *A*, the system admits an unstable behaviour shown in Fig. 5. Practically, it may happen that for a lower value of infected density due to a lower value of *k*, the fear about the disease fades away among the population and there is a minimal increase in the susceptible population for the lower value of constant recruitment. But in our assumption, infectious diseases always exist in our society, so due to the fearless behaviour of the population, which results in the instability of the system for the sudden increment, the system is unstable to the sudden increase in infected population.

Finally, we have compared the stability of the population for integer-order and fractional-order system. From Figs. 12 and 13, it is observed that when $r>\mu$, $S_{3}<S_{3}'$, i.e. the stability region for the fractional order is greater than the integer-order system and simultaneously $S_{4}>S_{4}'$ implies unstable region for fractional order is less than integer-order system. So it can be concluded that fractional-order model is more stable than integer-order model when $r>\mu$.

In the future, the model can be extended by considering vaccination, treatment, incorporating an incubation period and optimal control.

## Data Availability

All the materials and data included in this article are generated by the authors.
